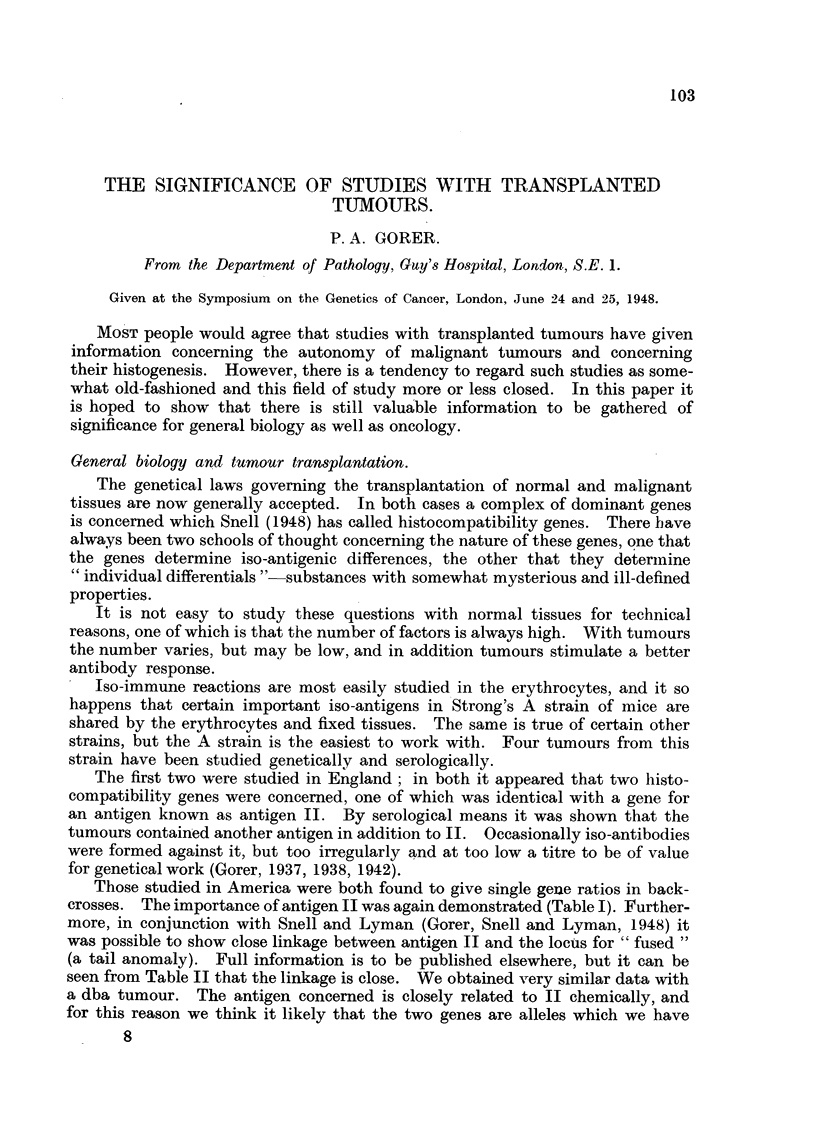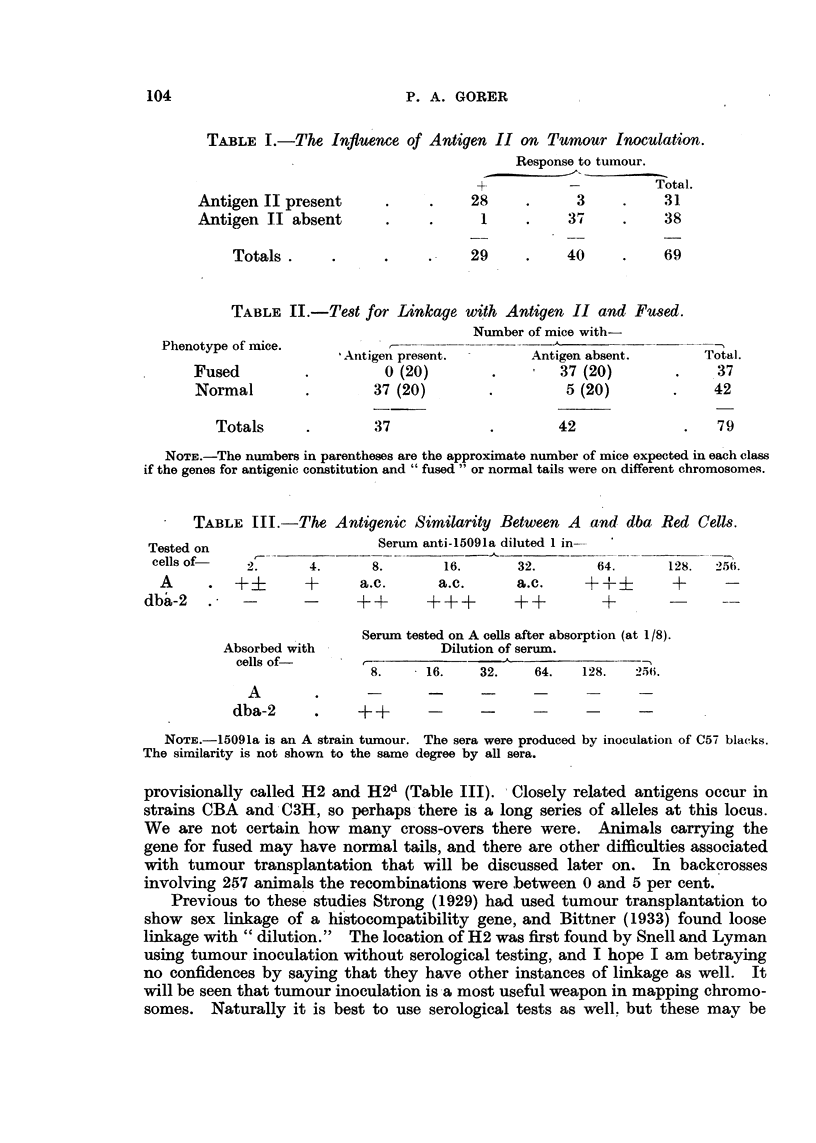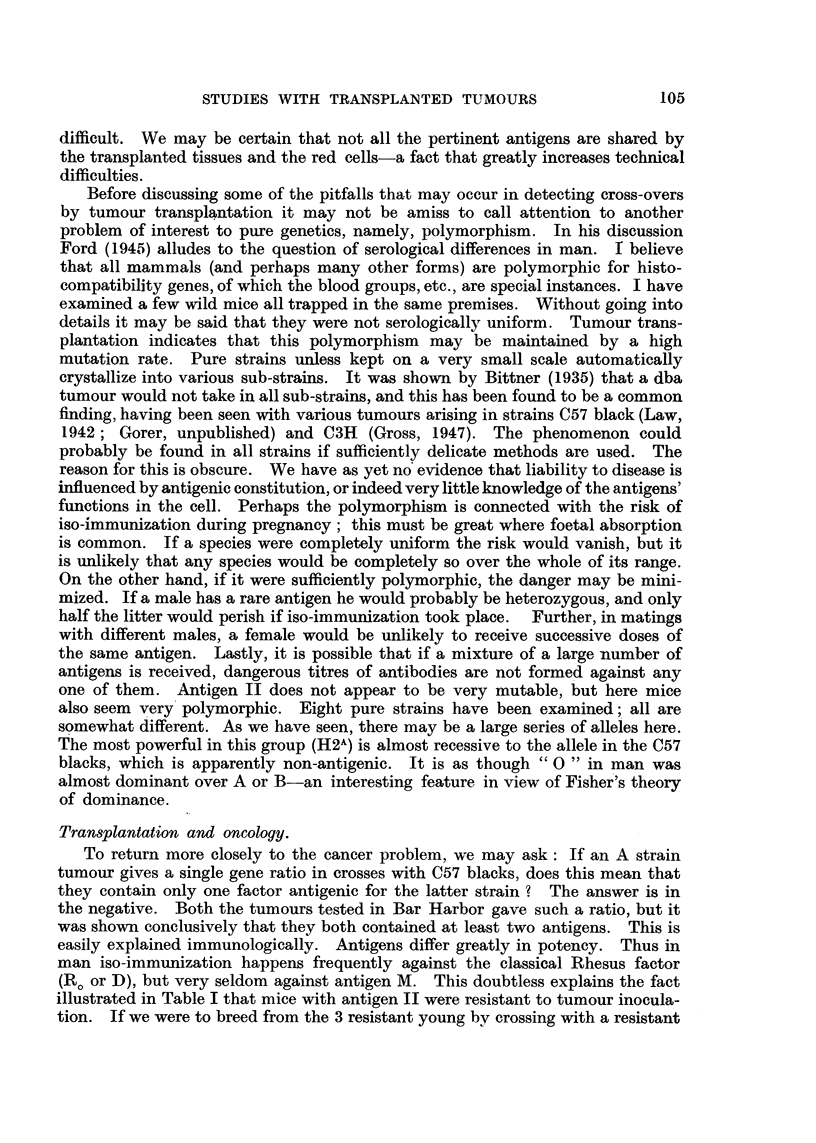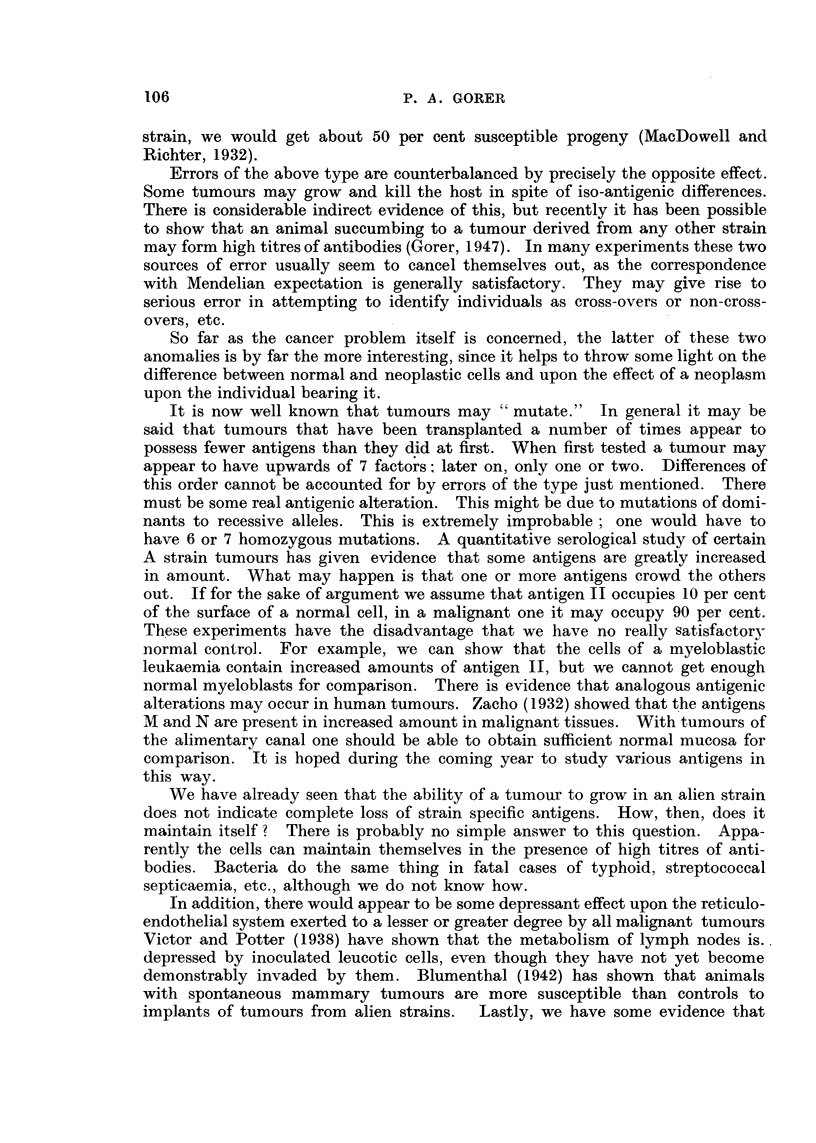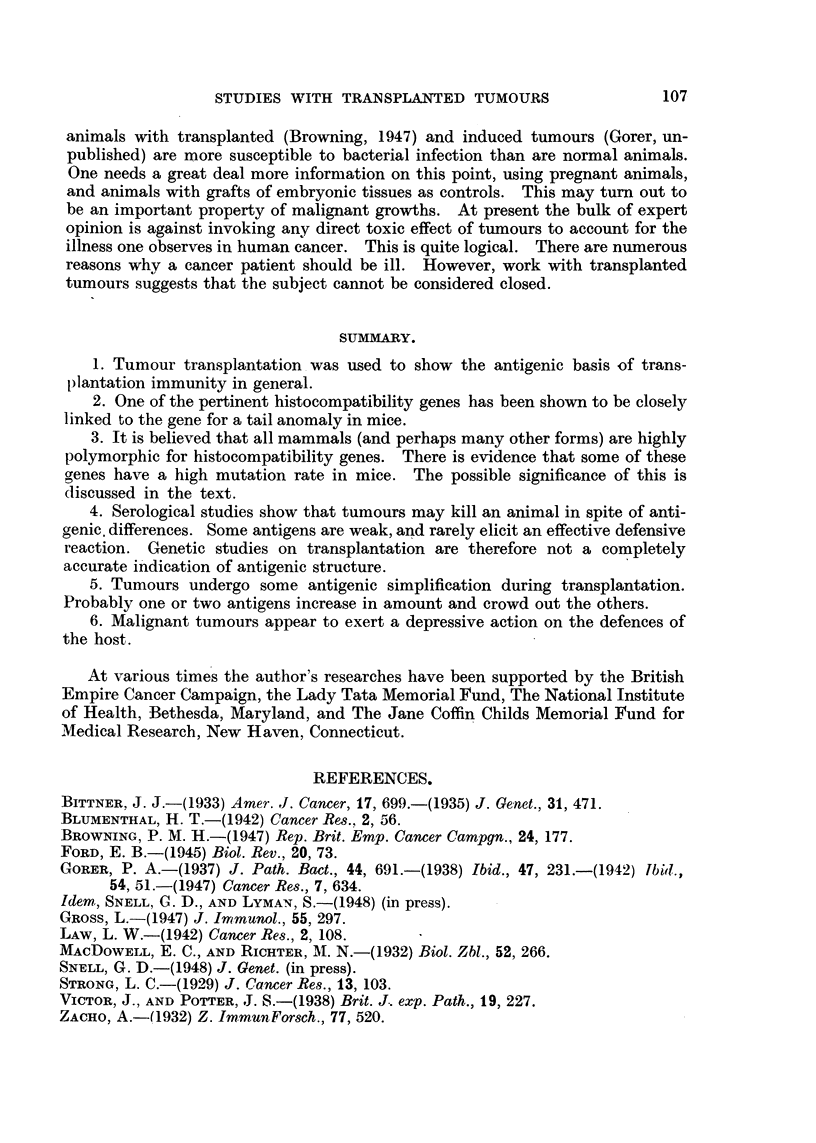# The Significance of Studies with Transplanted Tumours

**DOI:** 10.1038/bjc.1948.14

**Published:** 1948-06

**Authors:** P. A. Gorer


### THE SIGNIFICANCE OF STUDIES WITH TRANSPLANTED TUMOURS.

P. A. GORER.

*From the Department of Pathology, Guy's Hospital, London, S.E.* 1.

Given at the Symposium on the Genetics of Cancer, London, June 24 and 25, 1948.

MOST people would agree that studies with transplanted tumours have given information concerning the autonomy of malignant tumours and concerning their histogenesis. However, there is a tendency to regard such studies as somewhat old-fashioned and this field of study more or less closed. In this paper it is hoped to show that there is still valuable information to be gathered of significance for general biology as well as oncology.

*General biology and tumour transplantation.*

The genetical laws governing the transplantation of normal and malignant tissues are now generally accepted. In both cases a complex of dominant genes is concerned which Snell (1948) has called histocompatibility genes. There have always been two schools of thought concerning the nature of these genes, one that the genes determine iso-antigenic differences, the other that they determine "individual differentials"—substances with somewhat mysterious and ill-defined properties.

It is not easy to study these questions with normal tissues for technical reasons, one of which is that the number of factors is always high. With tumours the number varies, but may be low, and in addition tumours stimulate a better antibody response.

Iso-immune reactions are most easily studied in the erythrocytes, and it so happens that certain important iso-antigens in Strong's A strain of mice are shared by the erythrocytes and fixed tissues. The same is true of certain other strains, but the A strain is the easiest to work with. Four tumours from this strain have been studied genetically and serologically.

The first two were studied in England ; in both it appeared that two histocompatibility genes were concerned, one of which was identical with a gene for an antigen known as antigen II. By serological means it was shown that the tumours contained another antigen in addition to II. Occasionally iso-antibodies were formed against it, but too irregularly and at too low a titre to be of value for genetical work (Gorer, 1937, 1938, 1942).

Those studied in America were both found to give single gene ratios in backcrosses. The importance of antigen II was again demonstrated (Table I). Furthermore, in conjunction with Snell and Lyman (Gorer, Snell and Lyman, 1948) it was possible to show close linkage between antigen II and the locus for "fused" (a tail anomaly). Full information is to be published elsewhere, but it can be seen from Table II that the linkage is close. We obtained very similar data with a dba tumour. The antigen concerned is closely related to II chemically, and for this reason we think it likely that the two genes are alleles which we have

TABLE I.—*The Influence of Antigen II on Tumour Inoculation.*

| | Response to tumour. + | – | Total. |
|---|---|---|---|
| Antigen II present | 28 | 3 | 31 |
| Antigen II absent | 1 | 37 | 38 |
| Totals | 29 | 40 | 69 |

TABLE II.—*Test for Linkage with Antigen II and Fused.*

| Phenotype of mice. | Number of mice with— Antigen present. | Antigen absent. | Total. |
|---|---|---|---|
| Fused | 0 (20) | 37 (20) | 37 |
| Normal | 37 (20) | 5 (20) | 42 |
| Totals | 37 | 42 | 79 |

NOTE.—The numbers in parentheses are the approximate number of mice expected in each class if the genes for antigenic constitution and " fused " or normal tails were on different chromosomes.

TABLE III.—*The Antigenic Similarity Between A and dba Red Cells.*

| Tested on cells of— | Serum anti-15091a diluted 1 in— 2. | 4. | 8. | 16. | 32. | 64. | 128. | 256. |
|---|---|---|---|---|---|---|---|---|
| A | +± | + | a.c. | a.c. | a.c. | ++± | + | – |
| dba-2 | – | – | ++ | +++ | ++ | + | – | – |

| Absorbed with cells of— | Serum tested on A cells after absorption (at 1/8). Dilution of serum. 8. | 16. | 32. | 64. | 128. | 256. |
|---|---|---|---|---|---|---|
| A | – | – | – | – | – | – |
| dba-2 | ++ | – | – | – | – | – |

NOTE.—15091a is an A strain tumour. The sera were produced by inoculation of C57 blacks. The similarity is not shown to the same degree by all sera.

provisionally called H2 and H2$^d$ (Table III). Closely related antigens occur in strains CBA and C3H, so perhaps there is a long series of alleles at this locus. We are not certain how many cross-overs there were. Animals carrying the gene for fused may have normal tails, and there are other difficulties associated with tumour transplantation that will be discussed later on. In backcrosses involving 257 animals the recombinations were between 0 and 5 per cent.

Previous to these studies Strong (1929) had used tumour transplantation to show sex linkage of a histocompatibility gene, and Bittner (1933) found loose linkage with " dilution." The location of H2 was first found by Snell and Lyman using tumour inoculation without serological testing, and I hope I am betraying no confidences by saying that they have other instances of linkage as well. It will be seen that tumour inoculation is a most useful weapon in mapping chromosomes. Naturally it is best to use serological tests as well, but these may be difficult. We may be certain that not all the pertinent antigens are shared by the transplanted tissues and the red cells—a fact that greatly increases technical difficulties.

Before discussing some of the pitfalls that may occur in detecting cross-overs by tumour transplantation it may not be amiss to call attention to another problem of interest to pure genetics, namely, polymorphism. In his discussion Ford (1945) alludes to the question of serological differences in man. I believe that all mammals (and perhaps many other forms) are polymorphic for histocompatibility genes, of which the blood groups, etc., are special instances. I have examined a few wild mice all trapped in the same premises. Without going into details it may be said that they were not serologically uniform. Tumour transplantation indicates that this polymorphism may be maintained by a high mutation rate. Pure strains unless kept on a very small scale automatically crystallize into various sub-strains. It was shown by Bittner (1935) that a dba tumour would not take in all sub-strains, and this has been found to be a common finding, having been seen with various tumours arising in strains C57 black (Law, 1942 ; Gorer, unpublished) and C3H (Gross, 1947). The phenomenon could probably be found in all strains if sufficiently delicate methods are used. The reason for this is obscure. We have as yet no evidence that liability to disease is influenced by antigenic constitution, or indeed very little knowledge of the antigens' functions in the cell. Perhaps the polymorphism is connected with the risk of iso-immunization during pregnancy ; this must be great where foetal absorption is common. If a species were completely uniform the risk would vanish, but it is unlikely that any species would be completely so over the whole of its range. On the other hand, if it were sufficiently polymorphic, the danger may be minimized. If a male has a rare antigen he would probably be heterozygous, and only half the litter would perish if iso-immunization took place. Further, in matings with different males, a female would be unlikely to receive successive doses of the same antigen. Lastly, it is possible that if a mixture of a large number of antigens is received, dangerous titres of antibodies are not formed against any one of them. Antigen II does not appear to be very mutable, but here mice also seem very polymorphic. Eight pure strains have been examined; all are somewhat different. As we have seen, there may be a large series of alleles here. The most powerful in this group ($H2^A$) is almost recessive to the allele in the C57 blacks, which is apparently non-antigenic. It is as though " O " in man was almost dominant over A or B—an interesting feature in view of Fisher's theory of dominance.

*Transplantation and oncology.*

To return more closely to the cancer problem, we may ask : If an A strain tumour gives a single gene ratio in crosses with C57 blacks, does this mean that they contain only one factor antigenic for the latter strain ? The answer is in the negative. Both the tumours tested in Bar Harbor gave such a ratio, but it was shown conclusively that they both contained at least two antigens. This is easily explained immunologically. Antigens differ greatly in potency. Thus in man iso-immunization happens frequently against the classical Rhesus factor ($R_0$ or D), but very seldom against antigen M. This doubtless explains the fact illustrated in Table I that mice with antigen II were resistant to tumour inoculation. If we were to breed from the 3 resistant young by crossing with a resistant strain, we would get about 50 per cent susceptible progeny (MacDowell and Richter, 1932).

Errors of the above type are counterbalanced by precisely the opposite effect. Some tumours may grow and kill the host in spite of iso-antigenic differences. There is considerable indirect evidence of this, but recently it has been possible to show that an animal succumbing to a tumour derived from any other strain may form high titres of antibodies (Gorer, 1947). In many experiments these two sources of error usually seem to cancel themselves out, as the correspondence with Mendelian expectation is generally satisfactory. They may give rise to serious error in attempting to identify individuals as cross-overs or non-cross-overs, etc.

So far as the cancer problem itself is concerned, the latter of these two anomalies is by far the more interesting, since it helps to throw some light on the difference between normal and neoplastic cells and upon the effect of a neoplasm upon the individual bearing it.

It is now well known that tumours may " mutate." In general it may be said that tumours that have been transplanted a number of times appear to possess fewer antigens than they did at first. When first tested a tumour may appear to have upwards of 7 factors; later on, only one or two. Differences of this order cannot be accounted for by errors of the type just mentioned. There must be some real antigenic alteration. This might be due to mutations of dominants to recessive alleles. This is extremely improbable ; one would have to have 6 or 7 homozygous mutations. A quantitative serological study of certain A strain tumours has given evidence that some antigens are greatly increased in amount. What may happen is that one or more antigens crowd the others out. If for the sake of argument we assume that antigen II occupies 10 per cent of the surface of a normal cell, in a malignant one it may occupy 90 per cent. These experiments have the disadvantage that we have no really satisfactory normal control. For example, we can show that the cells of a myeloblastic leukaemia contain increased amounts of antigen II, but we cannot get enough normal myeloblasts for comparison. There is evidence that analogous antigenic alterations may occur in human tumours. Zacho (1932) showed that the antigens M and N are present in increased amount in malignant tissues. With tumours of the alimentary canal one should be able to obtain sufficient normal mucosa for comparison. It is hoped during the coming year to study various antigens in this way.

We have already seen that the ability of a tumour to grow in an alien strain does not indicate complete loss of strain specific antigens. How, then, does it maintain itself ? There is probably no simple answer to this question. Apparently the cells can maintain themselves in the presence of high titres of antibodies. Bacteria do the same thing in fatal cases of typhoid, streptococcal septicaemia, etc., although we do not know how.

In addition, there would appear to be some depressant effect upon the reticulo-endothelial system exerted to a lesser or greater degree by all malignant tumours Victor and Potter (1938) have shown that the metabolism of lymph nodes is depressed by inoculated leucotic cells, even though they have not yet become demonstrably invaded by them. Blumenthal (1942) has shown that animals with spontaneous mammary tumours are more susceptible than controls to implants of tumours from alien strains. Lastly, we have some evidence that animals with transplanted (Browning, 1947) and induced tumours (Gorer, unpublished) are more susceptible to bacterial infection than are normal animals. One needs a great deal more information on this point, using pregnant animals, and animals with grafts of embryonic tissues as controls. This may turn out to be an important property of malignant growths. At present the bulk of expert opinion is against invoking any direct toxic effect of tumours to account for the illness one observes in human cancer. This is quite logical. There are numerous reasons why a cancer patient should be ill. However, work with transplanted tumours suggests that the subject cannot be considered closed.

### SUMMARY.

1. Tumour transplantation was used to show the antigenic basis of transplantation immunity in general.
2. One of the pertinent histocompatibility genes has been shown to be closely linked to the gene for a tail anomaly in mice.
3. It is believed that all mammals (and perhaps many other forms) are highly polymorphic for histocompatibility genes. There is evidence that some of these genes have a high mutation rate in mice. The possible significance of this is discussed in the text.
4. Serological studies show that tumours may kill an animal in spite of antigenic differences. Some antigens are weak, and rarely elicit an effective defensive reaction. Genetic studies on transplantation are therefore not a completely accurate indication of antigenic structure.
5. Tumours undergo some antigenic simplification during transplantation. Probably one or two antigens increase in amount and crowd out the others.
6. Malignant tumours appear to exert a depressive action on the defences of the host.

At various times the author's researches have been supported by the British Empire Cancer Campaign, the Lady Tata Memorial Fund, The National Institute of Health, Bethesda, Maryland, and The Jane Coffin Childs Memorial Fund for Medical Research, New Haven, Connecticut.

### REFERENCES.

Bittner, J. J.—(1933) *Amer. J. Cancer*, **17**, 699.—(1935) *J. Genet.*, **31**, 471.

Blumenthal, H. T.—(1942) *Cancer Res.*, **2**, 56.

Browning, P. M. H.—(1947) *Rep. Brit. Emp. Cancer Campgn.*, **24**, 177.

Ford, E. B.—(1945) *Biol. Rev.*, **20**, 73.

Gorer, P. A.—(1937) *J. Path. Bact.*, **44**, 691.—(1938) *Ibid.*, **47**, 231.—(1942) *Ibid.*, **54**, 51.—(1947) *Cancer Res.*, **7**, 634.

*Idem*, Snell, G. D., and Lyman, S.—(1948) (in press).

Gross, L.—(1947) *J. Immunol.*, **55**, 297.

Law, L. W.—(1942) *Cancer Res.*, **2**, 108.

MacDowell, E. C., and Richter, M. N.—(1932) *Biol. Zbl.*, **52**, 266.

Snell, G. D.—(1948) *J. Genet.* (in press).

Strong, L. C.—(1929) *J. Cancer Res.*, **13**, 103.

Victor, J., and Potter, J. S.—(1938) *Brit. J. exp. Path.*, **19**, 227.

Zacho, A.—(1932) *Z. ImmunForsch.*, **77**, 520.